# Key Techniques in Tissue Culture of Scape Explants from *Hemerocallis citrina*

**DOI:** 10.3390/plants14172761

**Published:** 2025-09-04

**Authors:** Ying Wang, Qi Wei, Yamei Zhang, Shaowen Zheng

**Affiliations:** Horticulture College, Shanxi Agricultural University, Taigu 030801, China; s20222246@stu.sxau.edu.cn (Y.W.); z20223273@stu.sxau.edu.cn (Q.W.); z20223347@stu.sxau.edu.cn (Y.Z.)

**Keywords:** *Hemerocallis citrina*, tissue culture, floral stalk explants

## Abstract

Datong in Shanxi Province, known as the “Daylily Capital of China,” still primarily relies on traditional propagation by division for daylily seedling production. Although traditional seedling propagation methods are simple and low-cost, they suffer from limitations such as low propagation efficiency, which restricts large-scale production. The application of tissue culture in seedling production not only enables rapid large-scale propagation but also helps maintain desirable genetic traits through virus elimination. This study aimed to establish an efficient in vitro regeneration system for *Hemerocallis citrina* ‘Datong Huanghua’ through optimization of key culture stages using scape explants. The results demonstrated that during the stages of callus induction, adventitious bud differentiation, and proliferation culture, the best results were achieved using MS medium supplemented with 3 mg/L zeatin (ZT) and 0.3 mg/L α-naphthylacetic acid (NAA), yielding a callus induction rate of 83.33%, an adventitious bud differentiation rate of 83.40%, and a proliferation coefficient of 4.05. For root induction, MS medium containing 0.25 mg/L indole-3-butyric acid (IBA) and 0.25 mg/L NAA resulted in an average of 4.7 roots per plantlet with a 100% rooting rate. In addition, endogenous hormone analysis showed that lower ABA/GA_3_ and ABA/ZR ratios in scape explants promoted callus formation during the induction and differentiation stages.

## 1. Introduction

*Hemerocallis citrina* Baroni, a perennial herbaceous plant belonging to the genus Hemerocallis (family Asphodelaceae, order Liliales) [1], is notable for its long productive cycle with high economic benefits [2]. Commonly referred to as daylily, forget-sorrow grass, or lemon daylily, it includes the cultivar ‘Datong Huanghua’, which is distinguished by its greater plant height and a well-developed root system with long and uniformly slender roots [3]. The scape branches apically, forming corymbose or paniculate inflorescences. Young scapes are green and turn slightly yellowish-green at maturity, reaching a length of 80–130 cm; each scape bears 20–30 flower buds [4].

*Hemerocallis citrina* has significant edible, medicinal, and ornamental value [5]. Its edible part is the flower bud, which is harvested in the bud stage. The buds can be eaten dried after sun-drying or in fresh form. Because of this, it is known as one of the “Four Great Mountain Delicacies (Vegetarian Treasures)” [6]. Daylilies are rich in lecithin, which is a key component of many cells throughout the body, including those in the brain. It is believed to enhance brain function and slow aging, earning it the title of “Brain-Nourishing Vegetable” [7]. As a plant used for both food and medicine, the Compendium of Materia Medica (Ben Cao Gang Mu) records that daylily can clear heat, promote diuresis, aid digestion, improve eyesight, and relieve melancholy [8]. From the perspective of Traditional Chinese Medicine (TCM), daylily also has multiple therapeutic effects, such as reducing swelling, increasing urination, fighting bacteria, and reducing inflammation. It is also used to help with conditions including dizziness, lower back pain, edema, insufficient milk production, and joint pain and swelling [9]. Flavonoids are one of the main medicinal components in daylilies. They can boost immunity, improve blood circulation to remove stasis, and help alleviate depression [10].

Current propagation methods for *Hemerocallis citrina* in commercial production mainly involve sexual reproduction (seeds) and asexual techniques such as division, rhizome cutting, and bud cutting [11]. Seedling plants from seeds exhibit robust root systems and strong environmental adaptability, but they have long propagation cycles and tend to show trait variation [12,13]. Rhizome cutting requires more technical skill. Damage to roots can occur during handling, which may increase the risk of disease [14]. Division propagation has long cycles and a low propagation rate, which can lead to virus accumulation and varietal degradation [15]. Plant tissue culture offers distinct advantages over traditional propagation methods, primarily in the following aspects: Firstly, the technique enables low-cost, sustainable, and scalable production of secondary metabolites, independent of geographical and climatic constraints [16]; secondly, the strict sterile culture conditions ensure the production of materials with high phytosanitary standards [17]; and most importantly, the method maintains a high degree of genetic uniformity [18]. Therefore, developing optimized propagation protocols is important for producing large quantities of high-quality tissue-cultured plantlets.

Plant tissue culture, also called in vitro culture, is a technique that artificially simulates the internal environment of plants to allow plant cells or tissues to survive, grow, and differentiate under sterile conditions [19]. It is an essential tool for plant regeneration, rapid propagation, and the production of pathogen-free plants. This method offers benefits such as fast multiplication, preservation of desirable maternal traits, and high production efficiency, while also saving labor and costs [20]. Plant regeneration under in vitro conditions can occur through two pathways: direct organogenesis and indirect organogenesis (callus-mediated regeneration) [21]. In direct organogenesis, explants (such as leaves or stem segments) directly form adventitious shoots or roots from existing meristems or differentiated cells without forming callus. In indirect organogenesis, explants first dedifferentiate into callus tissue, which then redifferentiates into organs under the guidance of plant growth regulators [22]. For *Hemerocallis citrina* tissue culture, commonly used media include Murashige and Skoog (MS), Gamborg’s B5 (B5), Chu’s N6 (N6), and Half-Strength Murashige and Skoog (1/2 MS) media. Plant growth regulators are added to the culture systems to control cell growth, tissue differentiation, and organ regeneration [23].

In the tissue culture of the daylily, commonly used plant growth regulators include auxins and cytokinins. Auxins, such as α-Naphthaleneacetic acid (NAA), Indole-3-acetic acid (IAA), Indole-3-butyric acid (IBA), and 2,4-Dichlorophenoxyacetic acid (2,4-D), mainly promote callus formation, cell dedifferentiation, and root differentiation [23]. Cytokinins, including 6-Benzylaminopurine (6-BA), Kinetin (KT), Thidiazuron (TDZ), and Zeatin (ZT), primarily stimulate bud induction, cell division, and expansion [24].

Current studies on the in vitro regeneration of daylily (*Hemerocallis citrina*) have primarily focused on explants such as leaves and rhizomes. Traditional propagation methods exhibit an infection rate as high as 58.7% [25]. In existing reports, scape explants have not yet overcome the issue of excessive callus proliferation caused by high concentrations of 6-BA (3 mg/L), and there is a lack of genotype-specific optimization protocols. This study aimed to establish an efficient and reproducible in vitro regeneration system for *Hemerocallis citrina* ‘Datong Huanghua’ using scape explants by optimizing key stages, including callus induction, adventitious bud differentiation, proliferation, and rooting. In this study, scape explants of the ‘Datong Huanghua’ cultivar were used, through which optimized protocols for each stage were developed. Additionally, endogenous hormone levels during the induction phase were analyzed. These results collectively provide a theoretical and experimental basis for the tissue culture system of *Hemerocallis citrina*.

## 2. Results

### 2.1. Callus Induction and Adventitious Bud Differentiation from Floral Scape Explants

**Figure 1:**
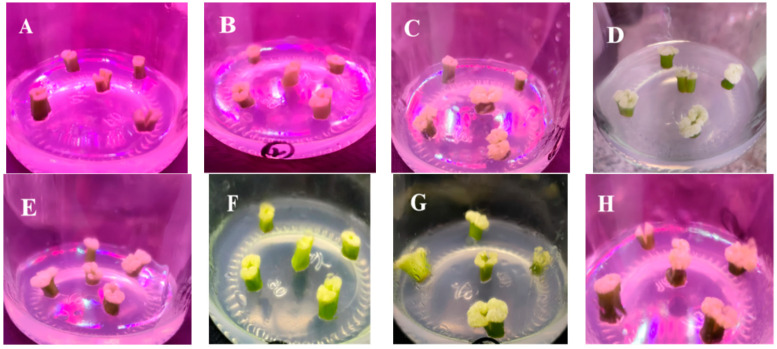
Callus induction status of floral scape explants under different treatments. (**A**) Scape explants showing callus formation on MS + 1.0 mg/L ZT; (**B**) Scape explants showing callus formation on MS + 1.0 mg/L ZT + 0.5 mg/L NAA; (**C**) Scape explants showing callus formation on MS + 2.0 mg/L ZT + 0.1 mg/L NAA; (**D**) Scape explants showing callus formation on MS + 2.0 mg/L ZT + 0.3 mg/L NAA; (**E**) Scape explants showing callus formation on MS + 3.0 mg/L ZT; (**F**) Scape explants showing callus formation on MS + 3.0 mg/L ZT + 0.1 mg/L NAA; (**G**) Scape explants showing callus formation on MS + 3.0 mg/L ZT + 0.3 mg/L NAA; (**H**) Scape explants showing callus formation on MS + 3.0 mg/L ZT + 0.5 mg/L NAA.

After disinfection, the explants were inoculated into MS media with different hormone combinations and cultured in darkness. After about 7 days, swelling was observed at the base of the explants, followed by the development of white, snowflake-like callus. By day 30, most explants had produced well-developed white callus. At 1 mg/L *Zeatin* (ZT) (Treatments 1–4), the callus induction rate increased with higher *α-Naphthaleneacetic acid* (NAA) concentrations, but the amount of callus was limited, induction was slow, and the callus volume was small. In Treatments 5–8, the induction rate first increased and then decreased. Among these, Treatments 6 and 7 produced more callus with better growth (Figure 1C,D). When ZT was used alone (Treatments 9–12), callus induction was less efficient than with ZT-NAA combinations. At 3 mg/L ZT (Figure 1F–H), all treatments showed higher induction rates, larger callus volumes, and stronger growth. Specifically, at 1 mg/L ZT, NAA concentrations from 0.0 to 0.5 mg/L did not significantly affect the induction rate. At 3 mg/L ZT, increasing NAA from 0.0 to 0.5 mg/L steadily improved the induction rate, reaching a maximum of 83% (Table 1). Overall, the best callus induction was achieved using MS medium with 3 mg/L ZT and 0.3 mg/L NAA.

**Figure 2:**
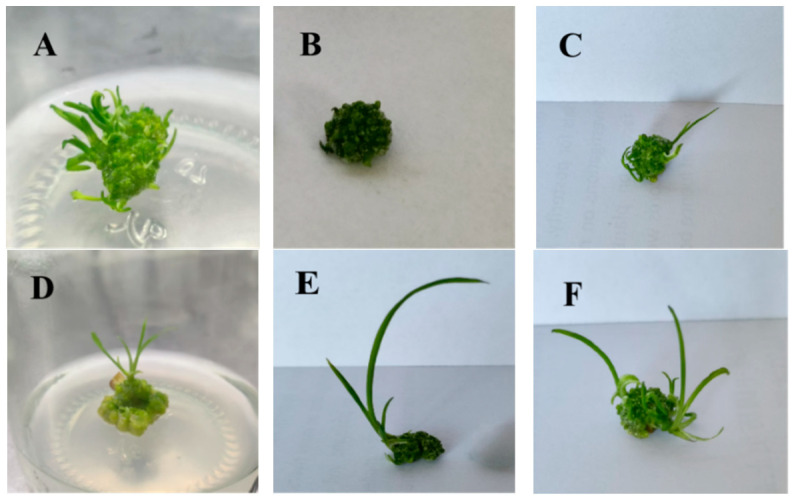
Adventitious bud differentiation in floral stalk explants under different treatments. (**A**) Shoot regeneration from scape callus on MS + 3.0 mg/L 6-BA + 0.1 mg/L NAA; (**B**) Shoot regeneration from scape callus on MS + 3.0 mg/L 6-BA + 0.3 mg/L NAA; (**C**) Shoot regeneration from scape callus on MS + 3.0 mg/L 6-BA + 0.5 mg/L NAA; (**D**) Shoot regeneration from scape callus on MS + 3.0 mg/L ZT + 0.1 mg/L NAA; (**E**) Shoot regeneration from scape callus on MS + 3.0 mg/L ZT + 0.3 mg/L NAA; (**F**) Shoot regeneration from scape callus on MS + 3.0 mg/L ZT + 0.5 mg/L NAA.

After 14 days of light culture following the transfer from darkness, the white callus gradually turned green and developed multiple protuberances, marking the start of adventitious bud differentiation. The combinations of 6-BA, ZT, and NAA significantly influenced the adventitious bud induction rate. At 3 mg/L *6-Benzylaminopurine* (6-BA) (Treatments 1–3), higher NAA concentrations resulted in lower induction rates. Treatment 1 produced a large number of clustered shoots with high leaf expansion and broad leaves. Treatment 2 had sparse adventitious buds, and Treatment 3 showed fewer growing points, moderate leaf expansion, and elongated leaves, indicating that high NAA levels inhibit bud differentiation (Figure 2B,C). With 3 mg/L ZT (Treatments 4–6), as NAA increased from 0.1 to 0.5 mg/L, the differentiation rate first increased and then decreased. The highest rate, 83.4%, occurred with 0.3 mg/L NAA (Treatment 5). Treatment 4 produced fewer adventitious buds with pale green, narrow leaves (Figure 2D). Treatment 5 developed larger, dark green leaves (Figure 2E). Although Treatment 6 had many growing points, high leaf expansion, and green coloration (Figure 2F), its lower differentiation rate made it less suitable for further experiments (Table 2). In summary, the optimal medium for adventitious bud differentiation was MS supplemented with 0.3 mg/L NAA and 3 mg/L ZT.

### 2.2. Multiplication Culture of Scape Explants

**Figure 3:**
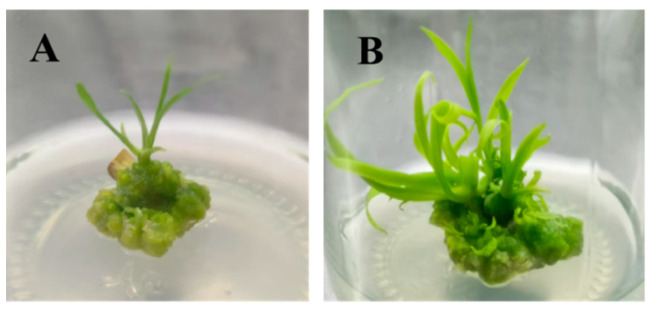
Proliferation effects of adventitious shoots from floral stalk explants. (**A**) Scape explants before proliferation culture on MS medium; (**B**) Scape explants at day 30 of proliferation culture on MS medium.

The induced adventitious shoots (including both clustered and single shoots) were cultured for proliferation. The multiplication coefficients ranged from 2.25 to 4.05 across treatments (Table 3). At 3 mg/L 6-BA, the multiplication coefficient decreased as the NAA concentration increased. At 0.5 mg/L NAA, it reached the lowest value of 2.45, with pale green leaves and very little yellowish-green callus formed at the base. In contrast, at 0.1 mg/L NAA, the multiplication coefficient was 3.65, and the leaves were green with yellowish-green callus at the base. The medium with 3 mg/L ZT and 0.3 mg/L NAA showed the best proliferation results, with a maximum multiplication coefficient of 4.05. The shoots grew vigorously, with significantly increased clusters, dark green leaves, and mostly green callus with some pale yellow areas at the base (Figure 3). This result was significantly better than that of the 0.5 mg/L NAA treatment. These findings show that the best medium for adventitious shoot proliferation was MS basal medium with 3 mg/L ZT and 0.3 mg/L NAA (Figure 3B).

### 2.3. Rooting Culture of Tissue-Cultured Plantlets Derived from Floral Stalks

**Figure 4:**
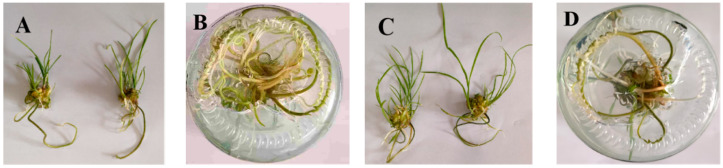
Rooting treatment of floral stalk-derived plantlets. (**A**,**B**) Rooting was induced in scape explants on MS medium containing 0.25 mg/L IBA and 0.25 mg/L NAA over 30 days; (**C**,**D**) Rooting was induced in scape explants on MS medium containing 0.50 mg/L IBA and 0.50 mg/L NAA over 30 days.

Plantlets derived from floral stalks showed high rooting rates (98.0–100.0%) when cultured on MS medium with different concentrations of IBA and NAA, indicating that MS basal medium supports efficient rooting (Table 4). The medium with 0.25 mg/L IBA and 0.25 mg/L NAA resulted in 100% rooting. The root systems were well-developed, with an average of 4.7 roots per plantlet. The roots had strong primary roots and thin lateral roots, indicating optimal growth (Figure 4). Overall, the best rooting medium was MS basal medium with 0.25 mg/L IBA and 0.25 mg/L NAA (Figure 4A,B).

### 2.4. Dynamic Changes in Endogenous Phytohormone Levels During In Vitro Induction Culture of Floral Scape Explants

#### 2.4.1. Changes in Zeatin Riboside (ZR), Gibberellic Acid (GA_3_), Indole-3-Acetic Acid (IAA), and Abscisic Acid (ABA) Contents

**Figure 5:**
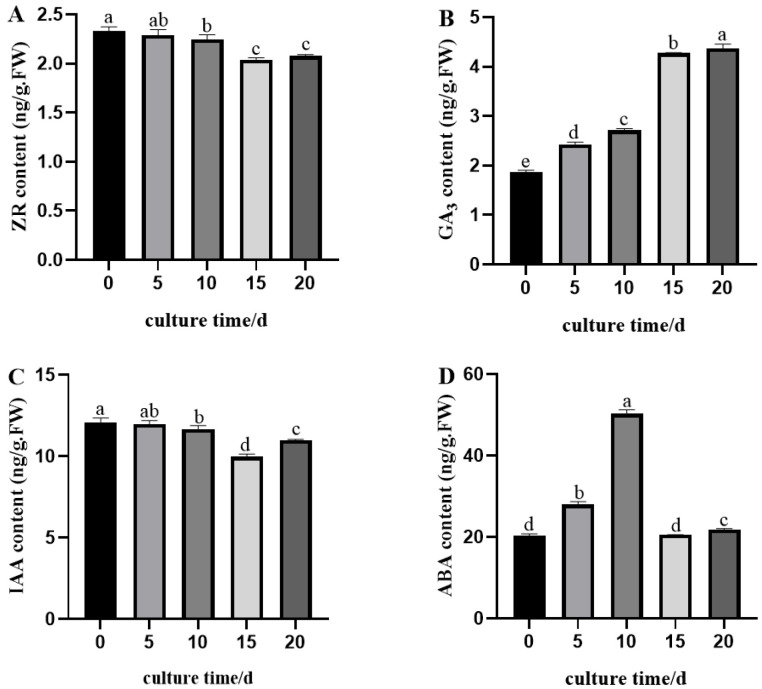
Dynamic changes in endogenous phytohormone levels during in vitro induction culture of floral scape explants. (**A**) Changes in ZR content during induced culture; (**B**) Changes in GA_3_ content during induced culture; (**C**) Changes in IAA content during induced culture; (**D**) Changes in ABA content during induced culture. The data in the figure are all “mean ± standard deviation”, and different lowercase letters indicate significant differences between treatments (*p* < 0.05, analysis of variance).

Analysis of the dynamic changes in endogenous hormones (ZR, GA_3_, IAA, and ABA) in scape explants during induction culture (Figure 5) showed the following results: The ZR content first decreased and then increased slightly, but declined overall. The GA_3_ content increased continuously. The IAA content first decreased and then increased. The ABA content first rose and then fell. Specifically, GA_3_ increased steadily from day 0 to 20, reaching a peak of 2.49 ng/g (Figure 5B). This suggests that higher GA_3_ levels improve induction efficiency and support callus growth. ZR was initially the highest (2.33 ng/g) but gradually decreased to 1.92 ng/g by day 15 before increasing again. This indicates that early ZR consumption promotes cell division and callus formation. IAA decreased to 9.98 ng/g by day 15 and then increased. This implies that the initial drop in IAA helps callus induction, while the later rise supports cell division (Figure 5C). ABA (Figure 5D) increased slowly from day 0 to 5, then rapidly to 50.26 ng/g by day 10, before dropping sharply and stabilizing after day 15. This shows that lower ABA levels favor cell division and proliferation, while helping maintain callus growth.

#### 2.4.2. Changes in Endogenous Hormone Ratios

**Figure 6:**
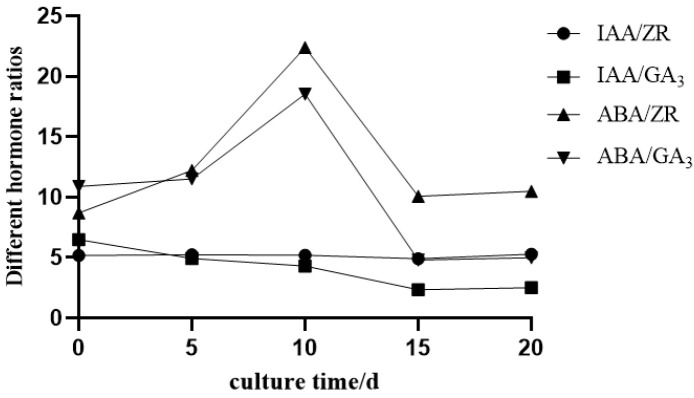
Dynamic changes in the ratios of four phytohormones (ABA, GA_3_, IAA, ZR) in floral stalk-derived explants.

Analysis of hormone ratios (Figure 6) showed that both the ABA/ZR and ABA/GA_3_ ratios first increased and then decreased, with significant fluctuations. In contrast, the IAA/GA_3_ ratio continued to decline with only minor changes, and the IAA/ZR ratio remained relatively stable. Specifically, from day 0 to 10, the ABA/GA_3_ ratio consistently increased, which helped start cell dedifferentiation and created favorable conditions for callus formation. As callus development progressed (10–15 d), the ABA/GA_3_ ratio decreased rapidly. This lower ratio promoted further callus differentiation and growth. The ABA/ZR ratio showed a trend similar to that of ABA/GA_3_. The decrease in the IAA/GA_3_ ratio suggested a weaker promotion of cell division, which was not beneficial for callus induction. The stable IAA/ZR ratio indicated that IAA-mediated cell elongation and ZR-mediated cell division were balanced during induction, thus supporting normal callus growth and proliferation.

## 3. Discussion

In this study, an efficient regeneration system based on scape explants was established. By optimizing the combination of explant growth regulators (6-BA:NAA = 6:1) and combining the dynamic analysis of endogenous hormones, the significant correlation between IAA/CTK ratio and flower stem regeneration efficiency was revealed for the first time (*p* < 0.01), providing a new strategy for establishing a genotype-specific regeneration system.

Daylily has a history of 2700 years since it was eaten as a delicacy, treated as a good medicine, and viewed as a famous flower [26]. ‘Datong Huanghua’ is the main variety in Datong City, Shanxi Province, China, and has extremely high economic and medicinal value [27]. Plant tissue culture can be used as a method for commercial cultivation of large-scale, uniform, disease-free seedlings and the development of new genotypes. Not only that, it ensures the production of healthy seedlings throughout the year in a limited space [21]. In selecting explants, more targeted choices are made according to the different characteristics of the plant, because the regeneration ability will vary depending on the explant [28]. The regeneration efficiency of a scape depends on its meristem distribution. Young scapes at early stages of bolting may have higher cell activity, but an improper development stage may easily lead to a decrease in regeneration rate.

Both direct organogenesis and indirect organogenesis are important regeneration methods in plant tissue culture. They have in common that they both start when cells in specific parts of the explant are activated, undergo cell division and differentiation, and finally form new organs or plants. However, the key difference between the two lies in whether they go through the callus stage. Direct organogenesis is the process in which explant cells differentiate directly into organs (such as buds or roots) without undergoing a callus stage. This method has the characteristics of high genetic stability and rapid regeneration [29]; Indirect organogenesis requires that the explant cells be completely dedifferentiated to form calli, and then redifferentiated into organs under hormone induction [30]. This process may lead to a higher risk of somatic mutation, but it is suitable for a wider range of plant materials. Eashan Mukherjee et al. [31] compared the reserpine content in directly and indirectly regenerated plants. They found that direct regeneration produced higher reserpine levels than callus-derived regeneration. During direct regeneration, cells develop in a defined direction and uniformly synthesize secondary metabolites. In contrast, callus exhibits a disorganized structure, with only some cells remaining active. This leads to inconsistent developmental pathways and heterogeneous metabolite production. Felipe de Jesús Romo-Paz et al. [32] studied indirect organogenesis from leaf explants of Physalis philadelphica on MS medium. They found that indirect organogenesis effectively promoted shoot regeneration. All leaves (except the first cotyledon) originated from buds. The scape is a derivative structure of the bud rather than an independent bud organ. Both structures achieved more efficient explant regeneration through indirect organogenesis. In this study, scape explants were induced to form callus, which subsequently differentiated into adventitious shoots. This process is classified as indirect organogenesis. In this experiment, callus was induced from the scape explants, and the callus further induced adventitious buds, a process known as indirect organogenesis.

Hormones have a positive effect on plant tissue culture, and appropriate hormone addition and optimal ratios are crucial [33]. Cytokinins play a key role in the culture of adventitious branches [34], but there have been few reports on the effect of ZT on callus induction and proliferation culture in daylily scapes. Raúl Vargas et al. [35] compared the regeneration rates of Physalis peruana cotyledons under different concentrations of ZT, MT, 6-BA, IBA, NAA, and 2,4-D, and found that the highest regeneration rate could reach 62.5% when the combination of 2 mg/L ZT + 2 mg/L MT + 0.5 mg/L IBA was used; Lei Lili [36] compared the sprouting rate of stem segments induced by kiwifruit ruda No. 6 in different concentrations of 6-BA with NAA and ZT with NAA. It was found that the sprouting rate reached 100.00% when cultured on the medium of MS + 0.5 mg/L ZT + 0.1 mg/L NAA + 3% sucrose for 60 days. Li Youli et al. [37] compared the induction rates of Quercus chinensis leaves under different concentrations of ZT, IAA, and 2,4-D, and found that the induction rate could reach 77.77% under the combination of 1.0 mg/L ZT + 0.05 mg/L IAA + 0.20 mg/L 2,4-D, with each callus having an average of 15.78 adventitious buds. Therefore, in this experiment, the effects of 6-BA with NAA and ZT with NAA at different concentrations on the induction of adventitious buds and proliferation culture stages were compared. It was found that with increasing ZT concentration, the rate of callus formation and adventitious bud induction first increased and then decreased, and the morphology of the callus also first improved and then deteriorated, indicating that too high or too low ZT concentration is not conducive to induction and culture. During the proliferation and culture stage, the scapes were tested with a combination of 6-BA with ZT and 6-BA with NAA. The results showed that ZT had a better effect during the test, which was reflected in the increase in the number of scapes calli and their green coloration. The proliferation coefficient also reached the highest value of 4.05.

In this study, scapes of ‘Datong Huanghua’ were used as explants. Through systematic optimization of plant growth regulator combinations, the regeneration efficiency was significantly improved. Compared with existing literature [38,39,40,41,42,43,44,45,46,47] (Table 5), notable breakthroughs were achieved in several key metrics: in proliferation efficiency, a proliferation coefficient of 4.05 was obtained, which is significantly higher than those reported in culture systems using the same explant type (e.g., although a higher coefficient of 6.67 was reported in study [47] using shortened stems, no rooting data were provided); in rooting ability, the use of a combined auxin formulation containing 0.25 mg/L IBA and 0.25 mg/L NAA increased the rooting rate to 100%, compared to the 90% rate achieved with 0.50 mg/L NAA alone as reported in references [39,41]. This result represents the highest rooting rate among all reported data, outperforming even those achieved with other explant types (e.g., 96% in shoot tips and 65% in leaves). The results indicate that scapes are an excellent explant source for the in vitro propagation of ‘Datong Huanghua’. The MS medium supplemented with 0.25 mg/L IBA and 0.25 mg/L NAA synergistically promoted adventitious shoot proliferation and root development, providing a reliable solution for large-scale seedling production of this cultivar.

Plant hormones are crucial endogenous regulators that control cell differentiation, developmental direction, and processes, and regulate and integrate plant growth [48]. During the induction phase, IAA primarily functions to induce callus formation, while ABA inhibits growth and promotes dormancy and senescence during plant growth and development [49]. In the tissue culture process of *Hemerocallis citrina*, the fluctuation amplitudes of ABA and IAA were large during the callus induction stage, indicating that higher levels of ABA and IAA, along with lower levels of ZR and GA_3_, are required for callus induction. ABA and IAA play primary roles in the process of callus induction. During the scape induction culture stage, the change in ZR content first decreased and then increased, showing an overall downward trend; the change in GA_3_ content exhibited a gradually increasing trend; the change in IAA content showed an initial decrease followed by an increase; ABA showed an initial increase followed by a decrease. The relatively high levels of IAA and ABA indicate that IAA and ABA play significant roles in the scape induction process.

## 4. Materials and Methods

### 4.1. Experimental Materials

The experiment was conducted at the Horticultural Research Center of Shanxi Agricultural University, located in Jinzhong City, Shanxi Province, China. This study used the cultivar ‘Datong Huanghua’ (*Hemerocallis citrina*, which refers to daylily produced in a specific area in Datong, Shanxi Province, China) as the experimental material. The plant samples were collected from a daylily cultivation base in Yunzhou District, Datong City, Shanxi Province, China (latitude 39°40′–40°16′ N, longitude 113°20′–113°55′ E). Sampling was conducted around 2:00 PM under clear weather conditions in mid-June. Young scapes (more than 20 cm in length) were collected from vigorous, disease-free plants that had not yet flowered.

### 4.2. Surface Disinfection of Explants

The *disinfection* method of this test is the combination of the standard process and specific optimization of this study [50]. Prior to conducting formal regeneration experiments, surface sterilization pretreatment was applied to the scape explants in order to obtain aseptic materials. Based on preliminary experimental screening, treatments involving 75% ethanol and sodium hypochlorite (NaClO) at various concentrations and durations were tested. The optimal sterilization protocol was determined as follows: treatment with 75% ethanol for 30 s, followed by 8% NaClO for 10 min. This approach resulted in lower contamination and browning rates, while promoting explant survival. Detailed data from the sterilization trials are provided in Supplementary Materials, Table S1.

### 4.3. Treatment Protocols for Callus Induction and Adventitious Shoot Differentiation from Scape Explants Using Different Phytohormone Ratios

In this experiment, MS medium was used for callus induction from scape explants. A total of twelve experimental treatments were established, consisting of different concentrations of ZT (1 mg/L, 2 mg/L, 3 mg/L) and NAA (0.1 mg/L, 0.3 mg/L, 0.5 mg/L). Additionally, six further treatments were set up: 6-BA (3 mg/L) combined with NAA (0.1 mg/L, 0.3 mg/L, 0.5 mg/L), and ZT (3 mg/L) combined with NAA (0.1 mg/L, 0.3 mg/L, 0.5 mg/L) to induce adventitious shoot differentiation. The ratios between the two hormones (either 6-BA: NAA or ZT: NAA) were 30:1, 10:1, and 6:1, respectively. All cultures were maintained in darkness under identical conditions. Each bottle was inoculated with 2 explants, with 5 bottles per treatment and 3 replicates per experiment. After 30 days, explant growth was observed, and the callus induction rate was calculated.

### 4.4. Adventitious Shoot Multiplication Culture from Scape Explants

High-quality explants showing good growth on the induction medium were selected and transferred to shoot proliferation media containing different plant growth regulator combinations. Five treatments were set up in MS medium: combinations of 6-BA (3 mg/L) with NAA (0.1 mg/L and 0.5 mg/L), and ZT (3 mg/L) with NAA (0.1 mg/L, 0.3 mg/L, and 0.5 mg/L). All cultures were maintained under the same light conditions. Each bottle contained 2 explants, with 4 bottles per treatment and 3 experimental replicates. After 30 days, growth was observed, and the proliferation coefficient was calculated.

### 4.5. Rooting Culture of Tissue-Cultured Plantlets Derived from Scape Explants

Well-growing rootless tissue-cultured plantlets were transferred to MS rooting medium supplemented with two different auxin combinations: 0.25 mg/L IBA + 0.25 mg/L NAA and 0.50 mg/L IBA + 0.50 mg/L NAA. Each treatment consisted of five bottles, with two plantlets per bottle, and was replicated three times. All cultures were maintained under light conditions. After 45 days, the rooting rate, number of roots per plantlet, and overall growth status were recorded.

### 4.6. Endogenous Phytohormone Quantification

During the induction culture process, day lily scape explants were sampled on days 0, 5, 10, 15, and 20, respectively, and the sampling time of each treatment group was uniformly arranged between 9 a.m. and 11 a.m. After each sampling, accurately weigh 0.5 g of the sample, immediately freeze it with liquid nitrogen, and store it in an ultra-low temperature refrigerator at −80 °C for the determination of endogenous hormone content. Enzyme-linked immunosorbent assay (ELISA) was used to quantitatively analyze zeatin riboside (ZR), gibberellic acid (GA_3_), indole-3-acetic acid (IAA), and abscisic acid (ABA) in scape explants. These analyses were conducted by Beijing Beinong Tianyi Biotechnology Co., Ltd. (Beijing, China). Among them, mouse monoclonal antigens and antibodies against ZR, GA_3_, IAA, and ABA, as well as horseradish peroxidase-labeled IgG, were provided by the Plant Hormone Research Institute of China Agricultural University [51]. The specific operations were carried out according to the method described by He (1993) [51]. The experiment was completed in a 96-well microtiter plate. Fresh plant material (0.5 g) that had been frozen in liquid nitrogen for 30 min and stored at −80 °C was weighed, 2 mL of sample extract was added, and the mixture was ground into homogenate under ice bath conditions and transferred to a 10 mL centrifuge tube; the mortar was rinsed multiple times with 2 mL of extract, the extracts were combined, mixed well, and let stand at 4 °C. Samples were ground in liquid nitrogen using a pre-cooled mortar and pestle. They were then extracted with 80% methanol (containing 1 mmol·L^−1^ butylhydroxytoluene as antioxidant) that had been cooled in an ice bath, and the extraction was continued overnight at 4 °C. The extract was collected by centrifugation at 12,000× *g* at 4 °C for 15 min. The residue was resuspended with the same pre-cooled extract. After standing at 4 °C for 1 h, it was centrifuged again under the same conditions. The two supernatants were combined and passed through a C18 Sep-Pak solid phase extraction column (Waters, Milford, MA, USA). The effluent was collected and dried under nitrogen, and the residue was dissolved in PBS buffer (0.01 mol·L^−1^, pH 7.4) for the determination of ZR, GA_3_, IAA, and ABA.

The microplate (Nunc, Roskilde, Denmark) was coated with synthetic conjugates of ZR, GA_3_, IAA, and ABA to ovalbumin in a 50 mmol·L^−1^ sodium bicarbonate buffer (pH 9.6) and incubated at 37 °C overnight. Non-specific binding sites were blocked by adding ovalbumin solution (10 mg·mL^−1^) to each well. After 30 min of reaction at 37 °C, standards, samples to be tested, and antibodies were added in turn, and the plate was incubated at 37 °C for 45 min. The anti-ZR, GA_3_, IAA, and ABA antibodies used were prepared according to the method of Weiler et al. [52]. Horseradish peroxidase-labeled goat anti-rabbit immunoglobulin was then added to each well and incubated at 37 °C for 1 h. After the reaction, o-phenylenediamine (OPD) substrate solution was added, and the plate was incubated at 37 °C in the dark for 15 min. The reaction was then stopped by adding 3 mol·L^−1^ sulfuric acid to each well. Absorbance was measured at 490 nm using an ELISA microplate reader (Model EL310, Bio-TEK, Winooski, VT, USA), and the ELISA data were calculated according to the method described by Weiler et al. [52].

### 4.7. Culture Conditions

This study used MS medium as the basal medium, containing 4.74 g/L MS powder, 6.5 g/L agar, and 30 g/L sucrose. The pH was adjusted to 5.8 using 1 mol/L NaOH or HCl, followed by sterilization at 121 °C for 20–25 min in an autoclave. The culture room maintained a photosynthetic photon flux density (PPFD) of 27.75–46.25 μmol/(m^2^·s) with a 16 h/d photoperiod at 25 ± 2 °C. During the cultivation period, if contamination is observed in the explants, promptly transfer the uncontaminated explants to fresh culture medium to maintain optimal growth conditions.

### 4.8. Data Processing

Statistical analyses were performed using ANOVA in Excel and SPSS 25.0, with Duncan’s test for significance determination (*p* < 0.05). Data visualization was conducted using GraphPad Prism 9.0.

Callus induction rate = (number of callus produced/total number of inoculations) × 100%

Adventitious bud induction rate = (number of callus producing adventitious buds/total callus cultured) × 100%

Adventitious bud proliferation rate = (Number of newly formed adventitious buds ÷ Number of initially cultured buds) × 100%

The rooting rate = (number of rooted adventitious shoots/total number of cultured adventitious shoots) × 100%.

## 5. Conclusions

In this experiment, scapes of ‘Datong Huanghua’ were used as explants. MS medium supplemented with 3 mg/L ZT and 0.3 mg/L NAA gave the best results, with a callus induction rate of 83.33% and an adventitious bud differentiation rate of 83.40%. The same medium also supported strong proliferation, with a coefficient of 4.05. For rooting, MS medium containing 0.25 mg/L IBA and 0.25 mg/L NAA achieved a 100% rooting rate and an average of 4.7 roots per plantlet, successfully producing tissue-cultured plantlets. This efficient regeneration system provides a useful method for large-scale production and can help meet market demand.

## Figures and Tables

**Table 1:** Effects of different hormone ratios on callus induction rate of floral scape explants.

Treatment	Plant Hormones		Number of Explants	Out of the Healing Number	Recovery Rate%
	ZT/(mg/L)	NAA/(mg/L)			
A1	1.0	0.0	60	9	12.05 ± 12.86 d
A2	1.0	0.1	60	11	20.83 ± 29.84 d
A3	1.0	0.3	60	15	25.00 ± 30.15 d
A4	1.0	0.5	60	17	29.17 ± 27.87 cd
A5	2.0	0.0	60	10	16.67 ± 30.77 d
A6	2.0	0.1	60	22	37.50 ± 40.59 cd
A7	2.0	0.3	60	25	41.67 ± 26.83 bcd
A8	2.0	0.5	60	20	33.33 ± 28.87 cd
A9	3.0	0.0	60	21	35.42 ± 36.08 cd
A10	3.0	0.1	60	35	58.33 ± 41.74 abc
A11	3.0	0.3	60	50	83.33 ± 32.57 a
A12	3.0	0.5	60	40	66.67 ± 38.92 ab

The data in the table are all “mean ± standard deviation”, and different lowercase letters indicate significant differences between treatments (*p* < 0.05, analysis of variance).

**Table 2:** Effects of different hormone ratios on the adventitious bud differentiation rate of floral stalk explants.

Treatment	Plant Hormones			Number of Explants	Induced Number	Adventitious Bud Induction Rate%
	6-BA/(mg/L)	NAA/(mg/L)	ZT/(mg/L)			
B1	3.0	0.1	0.0	30	20	66.80 ± 22.33 ab
B2	3.0	0.3	0.0	30	10	33.30 ± 35.21 c
B3	3.0	0.5	0.0	30	13	43.30 ± 31.75 bc
B4	0.0	0.1	3.0	30	11	36.60 ± 33.23 c
B5	0.0	0.3	3.0	30	25	83.40 ± 23.57 a
B6	0.0	0.5	3.0	30	15	50.10 ± 32.53 bc

The data in the table are all “mean ± standard deviation”, and different lowercase letters indicate significant differences between treatments (*p* < 0.05, analysis of variance).

**Table 3:** Effects of different hormone ratios on adventitious shoot proliferation from floral stalk explants.

Treatment	Plant Hormones			Number of Explants	Proliferation Coefficient	Growth Condition
	6-BA/(mg/L)	NAA/(mg/L)	ZT/(mg/L)			
1	3.0	0.1	0.0	20	3.65 ± 1.00 a	+++
2	3.0	0.5	0.0	20	2.45 ± 0.69 b	++
3	0.0	0.3	3.0	20	4.05 ± 1.07 a	++++
4	0.0	0.5	3.0	20	2.25 ± 0.49 b	++

Lowercase letters indicate significant differences (*p* < 0.05), with “+” representing the number of cluster buds.

**Table 4:** Effects of different hormone ratios on rooting of floral stalk-derived plantlets.

Treatment	Basic Medium	Plant Hormones		Rooting Rate%	Average Number of Roots
		IBA/(mg/L)	NAA/(mg/L)		
1	MS	0.25	0.25	100%	4.7
2	MS	0.50	0.50	98%	3.1

**Table 5:** Comparison of Tissue Culture Effects across Different Daylily Cultivars and Explant Types.

Research Materials	Type of Explant	Basic Culture Medium	Proliferation Coeffcient	Rooting Rate %	Source of Literature
Novel daylily cultivar ‘Red Baby’	Shoot tip	MS + 0.2 mg/LIBA	6.17	96%	[38]
‘Datong Huanghua’	Flowering stem	MS + 0.50 mg/LNAA		90%	[39]
‘Datong Huanghua’	Leaf blade	MS + 2 mg/L 6-BA + 0.5 mg/L IBA		65%	[40]
‘Datong Huanghua’	Scape	MS + 0.50 mg/LNAA		90%	[41]
‘Datong Huanghua’	Seed (stem of aseptic seedling)	1/2 MS + 0.2 mg/LNAA		45%	[42]
Taiwanese daylily cultivar ‘Taichung No. 6’	Shoot bud	Proliferation: MS + 6-BA 2.0 mg/L + NAA 0.1 mg/L Rooting: 1/2 MS + 0.05 mg/LNAA + 0.2 mg/L6-BA	3.2	95%	[43]
‘Alias Peter Parker’, ‘Sloam Gumdrop’, ‘Imprimatur’, along with 19 additional cultivars	Young inflorescence	MS		>95%	[44]
‘March Flower’ Daylily	Leaf blade	1/2MS + 0.5 mg/L NAA		93.33	[45]
Premium Xiamen Daylily	Rhizome	MS		96%	[46]
‘Datong Huanghua’	Shortened stem	MS + 1 mg/L6-BA + 0.1 mg/LNAA	6.67		[47]
This study (on ‘Datong Huanghua’)	Scape	MS + 0.25 mg/L IBA + 0.25 mg/L NAA	4.05	100%	

## Data Availability

No new data were created.

## Supplementary Materials

The following supporting information can be downloaded at: https://www.mdpi.com/article/10.3390/plants14172761/s1, Table S1: Effects of different disinfection treatments on floral scape explants.
