# Supplementary material for: Key Techniques in Tissue Culture of Scape Explants from Hemerocallis citrina
Source: Plants (Basel). 2025 Sep 4;14(17):2761. doi: 10.3390/plants14172761 (PMC12430602; doi:10.3390/plants14172761)
Supplement: Supplementary file 1 [file plants-14-02761-s001.zip › plants-3785237-supplementary.pdf]

Table S1. Effects of different disinfection treatments on floral scape explants

**Treatment**	**75% C_2H_5OH**	**5% NaClO/ min**	**8% NaClO/ min**	**10% NaClO/ min**	**Pollution rate/%**	**Browning rate/%**	**Survival rate/%**
1	0.5	5	0	0	23.10±15. 94a	13.20±17. 04c	63.70±10. 44ab
2	0.5	10	0	0	10.00±31. 62b	33.40±38. 59abc	56.60±41. 79ab
3	0.5	15	0	0	3.30±10. 44b	30.00±42. 92bc	66.70±41. 6ab
4	0.5	0	5	0	0.00±0. 00b	26.70±37. 92bc	73.30±37. 92a
5	0.5	0	10	0	3.30±10.44b	16.70±28.44bc	80.00±28.2a
6	0.5	0	15	0	0.00±0. 00b	33.30±47. 14abc	66.70±47. 14ab
7	0.5	0	0	5	6.70±21. 19b	36.70±48. 33abc	56.60±47. 31ab
8	0.5	0	0	10	0.00±0. 00b	56.70±49. 82ab	43.30±49. 82ab
9	0.5	0	0	15	3.30±10. 44b	73.30±43. 92a	26.70±43. 92b
